# A new species and additional records of *Lobrathium* Mulsant & Rey (Coleoptera, Staphylinidae, Paederinae) from China

**DOI:** 10.3897/zookeys.326.5970

**Published:** 2013-08-26

**Authors:** Wen-Rong Li, Cong-Chao Dai, Li-Zhen Li

**Affiliations:** 1 Department of Biology, College of Life and Environmental Sciences, Shanghai Normal University, Shanghai, 200234, P. R. China

**Keywords:** Coleoptera, Staphylinidae, Paederinae, *Lobrathium*, China, taxonomy, new species

## Abstract

*Lobrathium fuscoguttatum* **sp. n.** (type locality: Guangxi) is described and illustrated. The latest key to the *Lobrathium* species of mainland China is modified to include the new species. Additional data are provided for six previously described species.

## Introduction

According to a recent checklist provided by Assing (2012), 43 species of the genus *Lobrathium* Mulsant & Rey, 1878 were reported from China. Later, 13 additional species were described from mainland China (Assing 2013; Li et al. 2013; Li et al. 2013), thus raising the total number of species known from China to 56. In this paper we report a new species of *Lobrathium* (Guangxi), and additional locality data for six previously described species. Illustrations of the previously described species listed below are provided by Assing (2012), Li et al. (2013) and Li et al. (2013).

## Material and methods

The material treated in this study is deposited in the Insect Collection of Shanghai Normal University, Shanghai, China (**SNUC**).

Type labels are cited in their original spelling. A slash (/) is used to separate different labels. Type material bears the following type label: ‘HOLOTYPE [red] or PARATYPE [yellow], [genus name, species name], sp. n., [authors of the species], det. 2013.

The specimens were killed with ethyl acetate and then dried. Materials were stored in 75% ethanol; genitalia and small parts were embedded in Euparal on plastic slides that were attached to the same pin with the specimens.

Morphological studies were carried out using an Olympus SZX 16 stereoscope. A digital camera Canon EOS 7D with MP-E 65 mm Macro Photo Lens was used for the habitus photos. An Olympus CX31 microscope and a Canon G9 digital camera were used for the photos of small structures.

The measurements of various body parts are abbreviated as follows: BL length of the body from the labral anterior margin to the anal end; HL length of the head from the anterior margin of the frons to the posterior margin of the head; HW maximum width of the head; PL length of the pronotum along the midline; PW maximum width of the pronotum; EL length of the elytra from the anterior margin to the elytral posterior margin along the suture; EW maximum width of the elytra; AL length of the aedeagus from the apex of the ventral process to the base of the aedeagal capsule.

## Taxonomy

### Modified couplets of the key (Li et al. 2013) to the *Lobrathium* species of mainland China

**Table:** 

5a	♂: aedeagus 1.70–1.72 mm long, ventral process apically not bifid. Guangxi	5b
–	♂: aedeagus 1.35 mm long, ventral process apically bifid	6
5b	♂: ventral process of the aedeagus broader	*Lobrathium anatinum* Li & Li, 2013
–	♂: ventral process of the aedeagus slender	*Lobrathium fuscoguttatum* sp. n.

#### Lobrathium configens

Assing, 2012

http://species-id.net/wiki/Lobrathium_configens\according to Li et al 2013

##### Material examined

(5 ♂♂, 11 ♀♀). **China, Sichuan:** 4 ♂♂, 11 ♀♀, Erlang Shan, 1310 m, 01–VII–2009, Li leg. **Hubei:** 1 ♂, Muyu, Shennongjia, 05–VIII–2002, Li & Tang leg.

##### Comment.

Some specimens from Erlang Shan, Sichuan, have indistinct or very small reddish elytra spots.

#### Lobrathium demptum

Assing, 2012

http://species-id.net/wiki/Lobrathium_demptum\according to Li et al 2013

##### Material examined

(1 ♂, 4 ♀♀). **China, Zhejiang:** 1 ♂, 2 ♀♀, Anji County, Longwang Shan, 30°26'N, 119°26'E, 1050–1200 m, 13–V–2013, Chen et al. leg.; 2 ♀♀, Tianmu Shan, Gaoling, 800 m, 26–IV–2008, He & Tang leg.

##### Comment.

*Lobrathium demptum* had previously been recorded from Longwang Shan and Tianmu Shan, Zhejiang (Li et al. 2013).

#### Lobrathium fuscoguttatum sp. n.

http://zoobank.org/31EB556C-EF29-4E37-8066-710DEC726CB3

http://species-id.net/wiki/Lobrathium_fuscoguttatum

Figure 1

##### Type material

(8 ♂♂, 1♀). **Holotype**, ♂: “China, Guangxi, Lingui County, Anjiangping, 1700 m, 25°33'N, 109°55'E, 17–VII–2011, Peng Zhong leg. / Holotype ♂, *Lobrathium fuscoguttatum*, sp. n. Li et al., det. 2013”. **Paratypes**, 1 ♂: “China, Guangxi, Lingui County, Anjiangping, 1700 m, 25°33'N, 109°55'E, 17–VII–2011, Peng Zhong leg.”; 3 ♂♂: “China: Guangxi, Lingui County, Anjiangping, 1400–1700 m, 25°33'N, 109°56'E, 14–VII–2011, Peng Zhong leg.”; 3 ♂♂, 1 ♀: “China: Guangxi, Jinxiu County, Yinshan Station, 1200 m, 24°10'N, 110°13'E, 23–VII–2011, Peng Zhong leg.”

##### Description.

**Figure 1.**
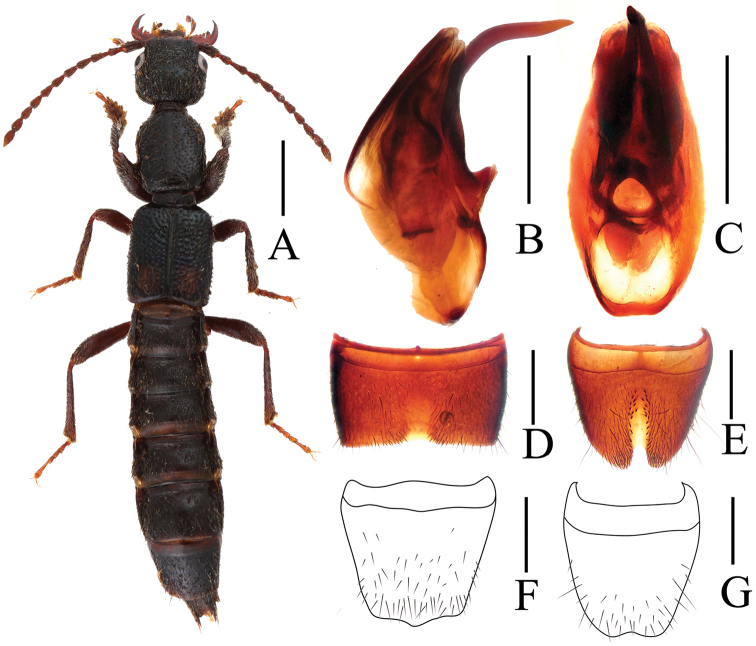
*Lobrathium fuscoguttatum*. **A** habitus **B** aedeagus in lateral view **C** aedeagus in ventral view **D** male sternite VII **E** male sternite VIII **F** female tergite VIII **G** female sternite VIII. Scales: **A** 1mm, **B**–**G** 0.5mm.

Body length 6.75–7.51 mm, length of fore body 3.45–3.89 mm. Habitus as in Fig. 1A. Coloration: body black with bluish hue, middle of elytra with yellowish spot not reaching lateral and posterior margins; legs blackish with paler tarsi, antennae dark brownish to blackish.

Head weakly transverse (HW/HL = 1.06–1.18), widest across eyes; posterior angles broadly rounded; punctation dense and moderately coarse, sparser in median dorsal portion; interstices without microsculpture. Eyes large, more than half as long as distance from posterior margin of eye to neck in dorsal view. Antenna 2.0–2.17 mm long.

Pronotum 1.19–1.27 times as long as broad, nearly as wide as head (PW/HW = 0.91–1.0), lateral margins convex in dorsal view, punctation similar to that of head, but with impunctate midline, interstices glossy.

Elytra wider, and nearly as long as pronotum (EL/EW = 0.91–1.04, EW/PW = 1.13–1.27, EL/PL = 0.92–1.01); punctation coarse and dense, arranged in series; interstices without microsculpture.

Abdomen broader than elytra; punctation fine and dense; posterior margin of tergite VII with palisade fringe.

**Male.** Sternite VII (Fig. 1D) strongly transverse and with distinct median impression, this impression without pubescence, posterior margin with distinct median concavity; sternite VIII (Fig. 1E) weakly transverse, with long and pronounced postero-median impression, this impression with a few modified, stout and short black setae (10-20 on either side of middle), posterior excision rather narrow and moderately deep, on either side of this excision with a cluster of long dark setae; aedeagus (Figs 1B, C) 0.82–0.87 mm long, ventral process long, curved, and apically acute in lateral view.

**Female.** Sternite VIII (Fig. 1G) weakly transverse, posteriorly convex; tergite VIII (Fig. 1F) posteriorly convex in middle.

##### Etymology.

The specific epithet (Latin, adjective) refers to the dark elytral spots.

##### Comparative notes.

This species is similar to *Lobrathium tortuosum* Li et al. (2013) in the shape and chaetotaxy of the sternites VII-VIII and the morphology of the aedeagus (the sternite VII of *Lobrathium tortuosum* see Li et al. 2013). The new species differs from *Lobrathium tortuosum* by the shape of the ventral process of the aedeagus in lateral view.

##### Habitat and distribution.

The specimens were sifted from wet moss near a cold stream in the Anjiangping National Reserve, Guangxi, in July (see fig. 20A in Li et al. 2013).

#### Lobrathium hebeatum

Zheng, 1988

http://species-id.net/wiki/Lobrathium_hebeatum\according to Li et al 2013

##### Material examined

(2 ♂♂, 1 ♀). **China, Henan:** 1 ♂, 1 ♀, Luoyang City, Baiyun Shan, 18–VII–2008, Li leg. **Ningxia:** 1 ♂, Jingyuan County, Erlonghe Forestry, Xiaonanchuan, Liangdianxia, 2000 m, 09–VII–2008, Yin leg.

##### Comment:

*Lobrathium hebeatum* was previously known only from Sichuan, Yunnan, Shaanxi and Gansu (Assing 2012; Assing 2013; Li et al. 2013). The above specimens represent the first records from Henan and Ningxia.

#### Lobrathium hongkongense

(Bernhauer, 1931)

http://species-id.net/wiki/Lobrathium_hongkongense\according to Li et al 2013

##### Material examined

(11 ♂♂, 1 ♀). **China, Fujian:** 1 ♂, Longyan City, Guihe Village, 1200 m, 25–V–2007, Huang & Xu leg. **Yunnan:** 1 ♂, 1 ♀, Nabanhe N. R., Chuguohe, Bengganghani, 1750 m, 28–IV–2009, Hu & Yin leg. **Zhejiang:** 2 ♂♂, Zhuji City, Dongbai Shan, 300 m, 29°28'N, 120°26'E, 04–X–2012, Zhao leg.; 1 ♂, Jingning County, Baiyunlin Village, 27°43'N, 119°39'E, 1100–1270 m, 07–V–2012, Zhu leg. **Hubei:** 6 ♂♂, Wufeng County, Houhe N. R., 1100 m, 30°04'N, 110°37'E, 27–IV–2004, Li leg.

##### Comment.

*Lobrathium hongkongense* is the most widespread species in China, and distributed also in southern Japan (Assing 2012; Assing 2013; Li et al. 2013).

#### Lobrathium quadrum

Li et al., 2013

http://species-id.net/wiki/Lobrathium_quadrum

##### Material examined

(1 ♂, 10 ♀♀). **China, Sichuan:** 1 ♂, 10 ♀♀, Dujiangyan City, Qingcheng Shan, 20–VII–2003, Li leg.

##### Comment.

The above specimens were collected from the type locality (Qingcheng Shan, Sichuan).

#### Lobrathium spathulatum

Assing, 2012

http://species-id.net/wiki/Lobrathium_spathulatum\according to Li et al 2013

##### Material examined

(2 ♂♂, 1 ♀). **China, Shaanxi:** 1 ♂, 1 ♀, Hanzhong City, Liping N. R., 1400–1600 m, 32°50'N, 106°36'E, 15–VII–2012, Chen et al. leg. **Jiangxi:** 1 ♂, Jinggangshan City, Ciping Town, 850 m, 26°29'N, 114°05'E, 18–X–2010, Peng et al. leg.

##### Comment.

This species has been recorded from Hubei, Shanxi, Zhejiang, Sichuan, Yunnan and Shaanxi (Assing 2012; Li et al. 2013). The above male from Jiangxi represents a new province record.

#### Lobrathium tortile

Zheng, 1988

http://species-id.net/wiki/Lobrathium_tortile\according to Li et al 2013

##### Material examined

(11 ♂♂, 5 ♀♀). **China, Shaanxi:** 8 ♂♂, 2 ♀♀, Hanzhong City, Liping N. R., 32°50'N, 106°36'E, 1400–1600 m, 15–VII–2012, Chen et al. leg.; 2 ♂♂, 1 ♀, Zhouzhi County, Houzhenzi, Qinling Shan, West Sangongli Valley, 33°50'N, 107°48'E, 18–V–2008, Xu leg.; 1 ♂, 2 ♀♀, Ankang City, Ningshaan County, Huoditang, 1500–1700 m, 12–VII–2012, Chen et al. leg.

##### Comment.

The specimens from Liping N. R., Hanzhong City, Shaanxi, were sifted from leaf litter near a stream after rain.

## Supplementary Material

XML Treatment for Lobrathium configens

XML Treatment for Lobrathium demptum

XML Treatment for Lobrathium fuscoguttatum

XML Treatment for Lobrathium hebeatum

XML Treatment for Lobrathium hongkongense

XML Treatment for Lobrathium quadrum

XML Treatment for Lobrathium spathulatum

XML Treatment for Lobrathium tortile
